# ﻿*Syntormon* Loew (Diptera, Dolichopodidae) from Inner Mongolia, China, with the description of a new species

**DOI:** 10.3897/zookeys.1212.119024

**Published:** 2024-09-16

**Authors:** Xingyang Qian, Chufei Tang, Ning Wang, Ding Yang

**Affiliations:** 1 Key Laboratory of Biohazard Monitoring and Green Prevention and Control in Artificial Grassland, Ministry of Agriculture and Rural Affairs, Institute of Grassland Research, Chinese Academy of Agricultural Sciences, Hohhot, Inner Mongolia 010010, China Ministry of Agriculture and Rural Affairs, Institute of Grassland Research, Chinese Academy of Agricultural Sciences Hohhot China; 2 Institute of Leisure Agriculture, Jiangsu Academy of Agriculture Sciences, Nanjing 210014, China Institute of Leisure Agriculture, Jiangsu Academy of Agriculture Sciences Nanjing China; 3 Department of Entomology, College of Plant Protection, China Agricultural University, Beijing 100193, China China Agricultural University Beijing China

**Keywords:** Barcoding, *Syntormondukha*, *Syntormonhenanense*, taxonomy

## Abstract

Previously, no records of *Syntormon* Loew, 1857 species were known from Inner Mongolia (China). The genus is reported here from Inner Mongolia for the first time, with the description of a new species, *S.sinicum***sp. nov.**, along with two previously described species, *S.dukha* Hollis, 1964 and *S.henanense* Yang & Saigusa, 2000. *Syntormonsinicum***sp. nov.** and *S.dukha* Hollis, 1964 are barcoded for the first time to support the species delimitation. A key to *Syntormon* species in China is provided.

## ﻿Introduction

The genus *Syntormon* Loew contains more than 110 known species worldwide (Yang et al. 2006; Grichanov 2014; Grichanov 2021). Species of *Syntormon* can be recognized in both sexes by the antenna pedicel with one short finger-like projection projecting into the postpedicel. In recent years, *Syntormon* has been included in several molecular phylogenetic studies which have shed light on its phylogenetic position. *Syntormon* belongs to Sympycninae, within the Dolichopodidae*sensu lato*, and the intergeneric relationships within the Sympycninae have been demonstrated (Bernasconi et al. 2007; Lim et al. 2010; Germann et al. 2011). There have been 63 mitochondrial sequences from 10 species reported in these studies, of which 23 and 10 sequences were sequenced from *Syntormonflexibile* Becker, 1922 and *S.pallipes* Fabricius, 1794, respectively. These data have supported the molecular identification of *S.pallipes* and *S.pseudospicatum* (Chursina and Grichanov 2019; Tonguç et al. 2023). Still, most *Syntormon* species can only be identified by morphological characteristics (e.g. Drake 2020, 2021).

Thus far, 15 known species of *Syntormon* are known to occur China, with four species recorded in the Palaearctic realm, nine species recorded in the Oriental realm, and two species recorded in both realms (Yang et al. 2011). The Palaearctic species are recorded from areas with a temperate continental climate, which is typical of Inner Mongolia, a province that belongs to Palaearctic China. The natural vegetation of Inner Mongolia encompasses a diverse range of ecosystems from forests, meadow steppe, steppe, desert steppe to Gobi Desert from northeast to southwest due to the greater precipitation in the northeast compared to the southwest and higher temperatures in the southwest compared to the northeast. However, there was no reports of *Syntormon* from Inner Mongolia.

The present study reports the distribution of *Syntormon* in Inner Mongolia for the first time, with records of a new species, *Syntormonsinicum* sp. nov., and two known species, *S.dukha* Hollis, 1964 and *S.henanense* Yang & Saigusa, 2000. This is also the first record of Sympycninae from Inner Mongolia. In addition, we provide the mitochondrial COI gene of *S.sinicum* sp. nov. and *S.dukha* Hollis, 1964 for the first time. A key to the species of the genus from China is provided.

## ﻿Materials and methods

### ﻿Morphological taxonomy

The specimens on which this study is based were collected in Inner Mongolia during 2013–2021 by sweep net. Hohhot, Bayan Nur, Baotou, Xilingol League, Ulanqab, Chifeng, Tongliao, Hinggan League in Inner Mongolia were investigated, except for Hulun Buir. We focused on wet biotopes such as river basins, lakes, and forests for collection. All specimens are deposited in the China Agricultural University (**CAU**), Beijing and Entomological Museum of Institute of Grassland Research, Chinese Academy of Agricultural Sciences (**IGRCAAS**). Morphological terminology follows Cumming and Wood (2017). Keys by Yang et al. (2011) for Chinese species were used to identify specimens collected. The following abbreviations are used: **acr** = acrostichal, **ad** = anterodorsal, **av** = anteroventral, **dc** = dorsocentral, **sc** = scutellars, **pd** = posterodorsal, **v** = ventral, **LI** = fore leg, **LII** = mid leg, **LIII** = hind leg, **CuAx ratio** = length of dm–cu / length of distal portion of CuA.

### ﻿DNA sequencing

Specimens used in this research were preserved in 95% ethanol at −20 °C. The mitochondrial genomic DNA was extracted from muscle tissue from thorax using the TIANamp Genomic DNA Kit (Tiangen) according to the manufacture’s protocol. All PCR reactions were performed in a 50 μL volume: 2 μL DNA extract, 25 μL Taq PCR Master Mix, 2 μL of each primer, and 19 μL ddH_2_O. The reaction cycle was set as follows: 94 °C for 5 min of initial degeneration, 35 cycles for 94 °C for 30 s, 56 °C for 30 s, 72 °C for 1 min, and a final extension of 72 °C for 10 min.

### ﻿Molecular identification

The newly sequenced and all available sequences of the mitochondrial COI gene of *Syntormon* were used to construct a distance-based neighbour-joining (NJ) phylogenetic tree, which was used for the species delimitation of the new species. Sequences that were significantly shorter than others, which were suspected to be incomplete, were pre-filtered. *Dolichopusornatipennis* Van Duzee, 1921 and *D.nigrilineatus* Van Duzee, 1924 were used as outgroups. Sequences are all available in the GenBank of National Center for Biotechnology Information (NCBI, https://www.ncbi.nlm.nih.gov) and the Barcode of Life Data System (BOLD, https://www.boldsystems.org/) (Table 1). CLUSTAL W was used to align the sequences (Kumar et al. 2018). The NJ tree was then constructed using the Kimura 2-patameter model and the 1000 rapid bootstrap replicates were performed using MEGA X (Kimura 1980; Trees 1987; Tamura et al. 2011).

**Table 1.** Sequences information used in molecular analysis.

Genus	Species	GenBank accession number
*Syntormon* Loew, 1857	*S.bicolorellum* Zetterstedt, 1843	MZ624427
	*S.dukha* Hollis, 1964	OR762505
	*S.flexibile* Becker, 1922	MG086951
	*S.freymuthae* Loew, 1873	MZ626600
	*S.pallipes* Fabricius, 1794	MZ611071
	*S.pumilum* Meigen, 1824	MZ608991
	*S.sinicum* sp. nov.	OR762504
	*S.tarsatum* Fallén, 1823	MZ628445
*Dolichopus* Latreille, 1796	*D.ornatipennis* Van Duzee, 1921	HM413216
	*D.nigrilineatus* Van Duzee, 1924	KC502345

## ﻿Taxonomy

### ﻿Key to the species of *Syntormon* from China

**Table:** 

1	First tarsomere of LIII with ventral appendages	**2**
–	First tarsomere of LIII without ventral appendages	**4**
2	Acr bristles uniseriate; 1^st^ tarsomere of LIII with 2 curved spines	***S.beijingense*** (Beijing)
–	Acr bristles biseriate; 1^st^ tarsomere of LIII with 4 irregular processes	**3**
3	Postpedicel 3.0 times longer than wide; hind tarsomere I with 2 same spines of same shape	***S.pallipes*** (Xinjiang, Qinghai, Beijing, Guizhou, Henan, Shaanxi)
–	Postpedicel less than 3.0 times longer than wide; hind tarsomere I with 2 differently shaped spines, 1 strong 1 thin (Fig. 1A)	***S.dukha*** (Yunnan, Inner Mongolia)
4	Hind tibia and tarsus flattened and plumose	***S.zhengi*** (Qinghai)
–	Hind tibia and tarsus simple	**5**
5	Hind tarsus slightly shortened, shorter than half length of tibia	**6**
–	Hind tarsus not shortened, longer than half length of tibia	**7**
6	First tarsomere of LI distinctly longer than length of tarsomeres II–V	***S.guizhouense*** (Guizhou)
–	First tarsomere of LI normal (not longer than tarsomeres II–V)	***S.flexible*** (Hebei, Fujian, Guangdong, Guizhou, Jiangsu, Zhejiang, Shanghai, Taiwan)
7	All coxae wholly black	**8**
–	Fore coxa yellow, mid and hind coxae black	**9**
8	Postpedicel 1.8 times longer than wide; arista 2.0 times longer than postpedicel; squama with black hairs	***S.luchunense*** (Yunnan)
–	Postpedicel 3.0 times longer than wide; arista distinctly shorter than postpedicel, no longer than 1/2 of postpedicel; squama with white hairs	***S.xizangense*** (Xizang)
9	Postpedicel rather short, about as long as wide (Fig. 1C, D)	***S.sinicum* sp. nov.** (Inner Mongolia)
–	Postpedicel distinctly elongated, more than 3.0 times longer than wide	**10**
10	Postpedicel 4.2 times longer than wide; arista 0.2 times as long as postpedicel	**11**
–	Postpedicel 3.5 times longer than wide; arista as long as postpedicel	**12**
11	Acr bristles 7–8 pairs; hind tibia with 1 ad brsitle and 5 pd bristles (Fig. 1B)	***S.henanense*** (Henan, Shaanxi, Yunnan, Inner Mongolia)
–	Acr bristles 13 pairs; hind tibia without ad bristles and with 2 or 3 pd bristles	***S.xinjiangense*** (Xinjiang)
12	Acr bristles 5 or 6 pairs; arista shorter than postpedicel	**13**
–	Acr bristles 20 in line, uniseriate; atista longer than postpedcel	***S.trisetum*** (Fujian)
13	Squama with yellowish hairs	**14**
–	Squama with black hairs	***S.luchunense*** (Yunnan, Guizhou)
14	Five dc bristles; 10 or 11 pairs of acr bristles	***S.medogense*** (Xizang)
–	Six dc bristles; 5 or 6 pairs of acr bristles	***S.emeiense*** (Sichuan, Guizhou)

#### ﻿ Syntormon dukha

Taxon classificationAnimaliaDipteraDolichopodidae

Hollis, 1964

9AA9F15E-C7D2-5BE2-B697-6F21B6BB81DF

Fig. 1A

Syntormon dukha Hollis, 1964: 93. Type locality: Nepal: Sangu, Taplejung.
Syntormon dukha Hollis, 1964. Yang et al. 2010: 1355.

##### Diagnosis.

Antenna postpedicel distinctly elongated, 3.0 times longer than wide; arista subapical, as long as postpedicel. First tarsomere of LI with 1 unequal bifurcated ventral spine at base.

##### Specimens examined.

China: Inner Mongolia, 5 males 5 females, Mount Helan, Huangqukou, 1900 m, 31.VII.2010, Yan Li (CAU-SYMSYN001A01-SYMSYN001A10); 1 male 1 female, Mount Jiufeng, Erdaogou, 1400–1500 m, 3.VIII.2013, Xiao Zhang (CAU-SYMSYN001B01-SYMSYN001B02); 10 males 8 females (OR762505), Mount Helan, Halawu, 2000 m, 5.VIII.2021, Xingyang Qian (IGRCAAS-SYMSYN1A01-SYMSYN1A18); 1 male 2 females, Hohhot, Xiaojinggou, 1400 m, 22.VIII.2021, Xingyang Qian (IGRCAAS-SYMSYN1B01-SYMSYN1B03); 1 male, Chifeng, Heilihe, 1000 m, 19.VII.2022, Xingyang Qian (IGRCAAS-SYMSYN1A01).

**Figure 1.**
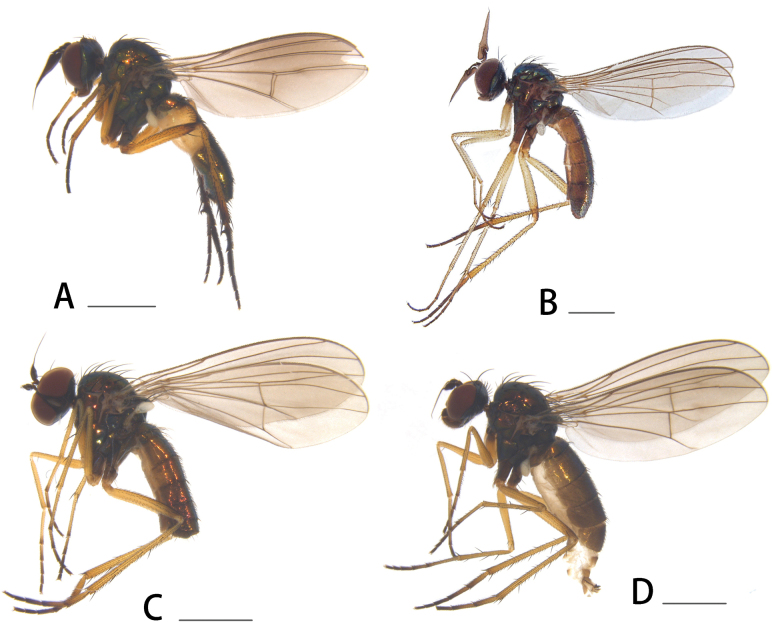
Habitus, lateral view **A***S.dukha* Hollis, 1964, male **B***S.henanense* Yang & Saigusa, 2000, male **C***S.sinicum* sp. nov., male **D***S.sinicum* sp. nov., female. Scale bars: 1 mm.

##### Habitat.

This species was collected in forests (Mount Jiufeng, Mount Helan, Xiaojinggou, Heilihe).

##### Distribution.

China (Yunnan, Inner Mongolia)

#### ﻿ Syntormon henanense

Taxon classificationAnimaliaDipteraDolichopodidae

Yang & Saigusa, 2000

E4D70999-7C0D-5EB3-A5AA-BC49376036F0

Fig. 1B

Syntormon henanense Yang & Saigusa, 2000: 207. Type locality: China: Henan, Songxian, Baiyunshan Mountain.
Syntormon henanense Yang & Saigusa, 2000. Yang et al. 2010: 1362.

##### Diagnosis.

Antenna postpedicel distinctly elongated; arista very short. 7 or 8 acr in a line, short-haired. Hind tibia with 1 antero-dorsal bristle and 5 postero-ventral bristles.

##### Specimens examined.

China: Inner Mongolia, 3 males, Mount Helan, Halawu, 13.VIII.2010, Yan Li (CAU-SYMSYN002A01-SYMSYN002A03); 2 males, Mount Jiufeng, Erdaogou, 1400–1500 m, 3.VIII.2013, Xiumei Lu (CAU-SYMSYN002B01-SYMSYN002B02).

##### Habitat.

This species was collected in forests (Mount Helan and Mount Jiufeng)

##### Distribution.

China (Henan, Shaanxi, Yunnan, Inner Mongolia).

#### ﻿ Syntormon sinicum sp. nov.

Taxon classificationAnimaliaDipteraDolichopodidae

7A1FD0C7-D409-5844-9555-7AE0ABF47F4B

https://zoobank.org/5C27BA90-3670-4A7A-9C2D-170B4C28D9E1

Figs 1C, D , 2A–D

##### Diagnosis.

Antennal scape with dorsal hairs; postpedicel 1.2 times longer than wide; arista long, much longer than postpedicel, subapical. Fore coxa yellow; mid and hind coxae black; hind femur brown at tip.

##### Description.

**Male.** Body length 3.1–3.3 mm, wing length 3.6–4.0 mm, based on three specimens.

**Figure 2.**
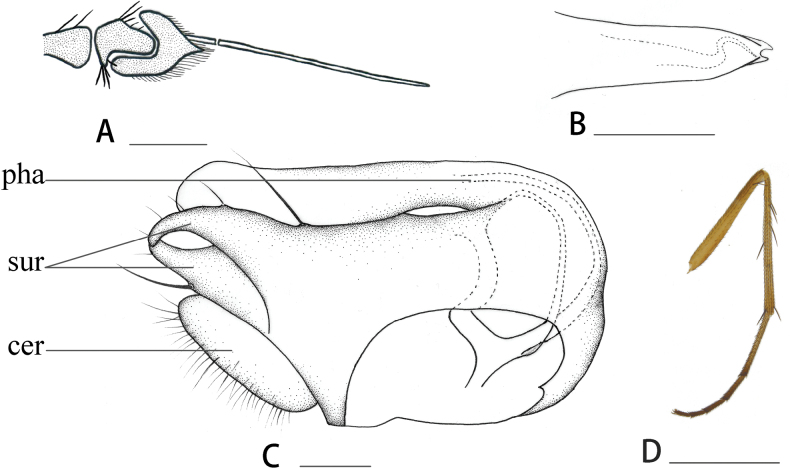
*Syntormonsinicum* sp. nov., male **A** antenna, lateral view **B** hypandrium, lateral view **C** genitalia, lateral view **D** mid leg, lateral view. Abbreviations: pha = phallus, sur = surstylus, cer = male cercus. Scale bars: 0.1 mm.

***Head*.** Frons wide, metallic green; face narrowed downwards, with gray pollinosity. Antenna (Fig. 2A) black; scape with 2 or 3 dorsal hairs; pedicel with 1 short, finger-like projection into postpedicel; postpedicel small, short, 1.2 times longer than wide; arista black, subapical, basal segment 0.15 times as long as apical segment. Proboscis and palpus dark brown, with black hairs, palpus with 1 blackish apical bristle.

***Thorax*.** Metallic green with pale gray pollinosity. Hairs and bristles on thorax black. 5 acr bristyles uniserate; 6 dc bristles, long and strong; scutellum with 2 strong bristles and 2 much smaller bristles. Propleuron with yellowish hairs.

***Legs*.** Mainly yellow; coxa of LI yellow, coxa of LI and LII black; trochanters of LII and LIII brownish yellow or dark brownish yellow; femur of LIII brown apically; tarsi of LI and LII dark brown from tip of tarsomere I onwards; leg of LIII brown to dark brown onwards entirely. Hairs and bristles on legs mostly black; coxa of LI with yellowish hairs and brown apical bristles, coxa of LII with brown hairs and bristles (1 strong long apical bristle dark brown), coxa of LIII with 1 blackish outer bristle. femora of LII and LIII each with 1 ad preapical bristle. tibia of LI with 1 pd bristle at middle and 2 short apical bristles; tibia of LII with 3 ad bristles, 1 pd bristle, 1 av bristle and 3 apical bristles; tibia of LIII with 3 ad bristles, 5 pd bristlesand 4 apical bristles. Relative lengths of tibia and 5 tarsomeres of legs LI: 1.95: 1.0: 0.5: 0.4: 0.3: 0.25; LII: 2.5: 1.2: 0.6: 0.45: 0.25: 0.2; LIII: 3.4: 0.8: 0.7: 0.5: 0.35: 0.3. Wing nearly hyaline; veins blackish, R_4+5_ and M slightly convergent apically, CuAx ratio 0.55. Squama yellow with brownish yellow hairs. Halter yellow.

***Abdomen*.** Metallic greenish with pale gray pollinosity. Hairs and bristles on abdomen mostly black, tergite 1 with yellow lateral hairs and bristles, hairs and bristles on tergites I–V yellow. Male genitalia (Fig. 2C): epandrium distinctly longer than wide; surstylus with dorsal lobe wide, lateral side with long bristles, ventral lobe very wide with apical bristles; male cercus blunt at tip.

**Female.** Body length 3.0–3.1 mm, wing length 3.6–4.4 mm based on 3 specimens. Postpedicel slightly short, as long as wide, face wide.

##### Molecular delimitation.

**Figure 3.**
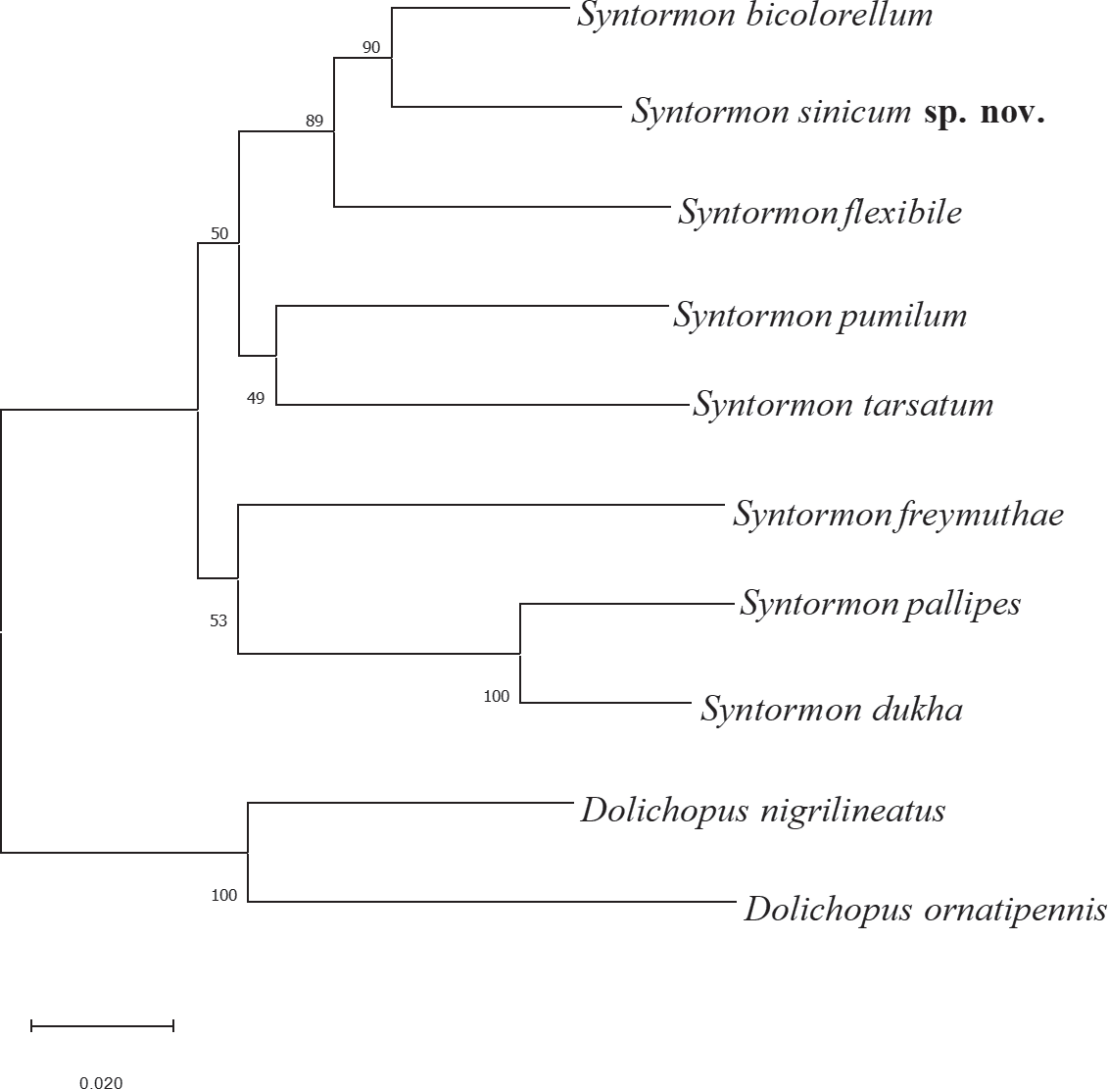
The neighbour-joining (NJ) tree of *Syntormon* species based on 10 mitochondrial COI sequences.

In the NJ phylogenetic tree, *S.sinicum* sp. nov. is sister to *S.bicolorellum* Zetterstedt, 1843, forming a clade with *S.flexible* Becker, 1922 (Fig. 3). The interspecific genetic distance between *S.sinicum* sp. nov. and other *Syntormon* species ranged from 5.72% to 20.47% (Table 2). These results support the classification of *S.sinicum* as a separate species (Hebert et al. 2003).

**Table 2.** Interspecific genetic distance between *Syntormon* COI sequences.

	*S.bicolorellus*	*S.flexibile*	*S.freymuthae*	*S.pallipes*	*S.pumilum*	*S.tarsatus*	*S.sinicum* sp. nov.	*S.dukha*	*D.nigrilineatus*	*D.ornatipennis*
***S.bicolorellus***										
***S.flexibile***	0.0805									
***S.freymuthae***	0.1258	0.1530								
***S.pallipes***	0.1330	0.1451	0.1388							
***S.pumilum***	0.1131	0.1205	0.1293	0.1420						
***S.tarsatus***	0.1062	0.1168	0.1459	0.1480	0.1132					
***S.sinicum* sp. nov.**	0.0572	0.0877	0.1277	0.1276	0.1203	0.1222				
***S.dukha***	0.1131	0.1356	0.1312	0.0542	0.1365	0.1460	0.1150			
***D.nigrilineatus***	0.1612	0.1668	0.1858	0.1726	0.1760	0.1760	0.1669	0.1923		
***D.ornatipennis***	0.1825	0.1936	0.2086	0.1904	0.1943	0.2058	0.2047	0.1985	0.1145	

##### Type material.

***Holotype***, male, China: Inner Mongolia, Mount Helan, Xiangchizi, 1900 m, 30.VII.2013, Xiao Zhang (CAU-SYMSYN003A01). ***Paratypes***, 2 females, same data as holotype (CAU-SYMSYN003B01); 5 males 5 females, Mount Helan, Xiangchizi, 1900 m, 7.VIII.2021, Liang Wang (CAU-SYMSYN003C01-SYMSYN003C10); 3 males 3 females (OR762504), Hohhot, Xiaojinggou, 1400 m, 21.VIII.2021, Xingyang Qian (IGRCAAS-SYMSYN2A01-SYMSYN2A06).

##### Habitat.

This species was collected in forests (Mount Helan, Xiaojinggou).

##### Distribution.

China (Inner Mongolia).

##### Remarks.

The new species is somewhat similar to *S.luchunense* Yang & Saigusa, 2001, but it can be distinguished by postpedicel, which is 1.2 times longer than wide. In *S.luchunense* Yang & Saigusa, 2001, the postpedicel is 2.5 times longer than wide (Yang et al. 2011). The new species is also somewhat similar to *S.brevicornis* Frey, 1936, but it has one long bristle on the ventral side of the mid tibia (Fig. 2D), and its fore tibia is yellow. In *S.brevicornis*, the mid tibia lacks ventral bristles, and the fore tibia is brown at the base (Frey 1936; Negrobov 1975).

## ﻿Discussion

**Figure 4.**
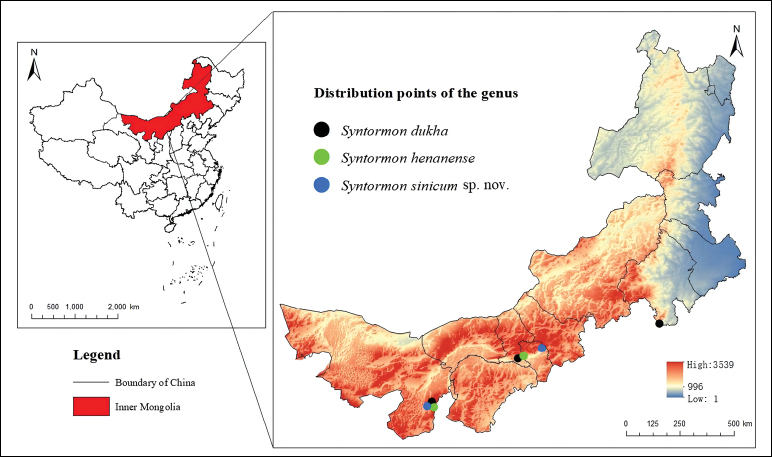
Distribution of S*yntormon* in Inner Mongolia.

The study reports *Syntormon* from Inner Mongolia for the first time. Records of the genus in Inner Mongolia are shown in Fig. 4. The province has a temperate continental climate over more than one million square kilometers, with a variety of natural landscapes, including forests, meadows, and grasslands, which is suitable for Dolichopodidae. There are several reports of the genus from the neighbouring regions, such as *S.flexibile* Becker, 1922 from Hebei province and *S.henanense* Yang & Saigusa, 2000 and *S.pallipes* Fabricius, 1794 from Shaanxi province. In addition, *S.beijingense* Yang, 1998 and *S.pallipes* Fabricius, 1794 are recorded from Beijing and *S.pallipes* Fabricius, 1794 and *S.xinjiangense* Yang, 1999 are recorded from Xinjiang province (Yang et al. 2011). Furthermore, the previous insect investigations in Inner Mongolia mainly focused on Mount Helan (Alax League), Mount Jiufeng (Baotou), and Saihanwula (XilinGol League), and were limited in scope. Therefore, the species diversity of Sympycninae in Inner Mongolia is undoubtedly underestimated.

## Supplementary Material

XML Treatment for Syntormon dukha

XML Treatment for Syntormon henanense

XML Treatment for Syntormon sinicum

## ﻿Additional information

### Author ORCIDs

Xingyang Qian https://orcid.org/0000-0002-5319-4510

Chufei Tang https://orcid.org/0000-0001-6266-4900

Ning Wang https://orcid.org/0000-0001-9377-5988

Ding Yang https://orcid.org/0000-0002-7685-3478
